# Antimicrobial Wound Dressings as Potential Materials for Skin Tissue Regeneration

**DOI:** 10.3390/ma12111859

**Published:** 2019-06-08

**Authors:** Andrei Paduraru, Cristina Ghitulica, Roxana Trusca, Vasile Adrian Surdu, Ionela Andreea Neacsu, Alina Maria Holban, Alexandra Catalina Birca, Florin Iordache, Bogdan Stefan Vasile

**Affiliations:** 1 Faculty of Applied Chemistry and Materials Science, Politehnica University of Bucharest, 060042 Bucharest, Romania; andrei93.paduraru@yahoo.com (A.P.); cghitulica@yahoo.com (C.G.); truscaroxana@yahoo.com (R.T.); adrian.surdu@upb.ro (V.A.S.); neacsu.a.ionela@gmail.com (I.A.N.); alina_m_h@yahoo.com (A.M.H.); ada_birca@yahoo.com (A.C.B.); floriniordache84@yahoo.com (F.I.); 2 National Centre for Micro and Nanomaterials, Politehnica University of Bucharest, 060042 Bucharest, Romania; 3 National Research Center for Food Safety, Politehnica University of Bucharest, 060042 Bucharest, Romania; 4 Microbiology Immunology Department, Faculty of Biology, University of Bucharest, 050095 Bucharest, Romania; 5 Faculty of Veterinary Medicine, University of Agronomic Science and Veterinary Medicine, 011464 Bucharest, Romania

**Keywords:** skin regeneration, zinc oxide nanostructures, antimicrobial properties, biocompatibility, electrospinning

## Abstract

The most important properties of performant wound dressings are biocompatibility, the ability to retain large amount of exudate and to avoid complications related with persistent infection which could lead to delayed wound healing. This research aimed to obtain and characterize a new type of antimicrobial dressings, based on zinc oxide/sodium alginate/polyvinyl alcohol (PVA). Zinc oxide nanostructures, obtained with different morphology and grain size by hydrothermal and polyol methods, are used as antimicrobial agents along with sodium alginate, which is used to improve the biocompatibility of the dressing. The nanofiber dressing was obtained through the electrospinning method. Characterization techniques such as X-ray diffraction (XRD) and scanning electron microscopy (SEM) were performed to determine the structural and morphological properties of the obtained powders and composite fibers. Their antimicrobial activity was tested against Gram negative *Escherichia coli* (*E. coli*), Gram positive *Staphylococcus aureus* (*S. aureus*) bacteria and *Candida albicans* (*C. albicans*) yeast strains. The in vitro biocompatibility of the obtained composites was tested on human diploid cells. The obtained results suggest that the composite fibers based on zinc oxide and alginate are suitable for antimicrobial protection, are not toxic and may be useful for skin tissue regeneration if applied as a dressing.

## 1. Introduction

Skin is the largest organ of the human body and acts as a protective barrier against pathogens and physical damage, and also has an important role in preventing excessive water loss from the body. Moreover, it is known that when the skin suffers physical trauma, a series of processes are initiated, involving the interactions between cells and matrix components, in order to restore its proper functioning. Damaged skin could be covered with a dressing that is designed to maintain a moist environment, helping the exchange process between the exterior and the wound, so that the oxygen reaches the affected skin, leading to a faster wound healing. Moreover, the properties of the materials used to obtain the dressing should promote the absorption of excess fluids [1,2]. Although the gaze wound dressing is still the most used in hospitals for minor injuries, the advancement of technology leads to a new approach to treat serious injuries, such as burns or infected wounds, using dressings in different forms depending on the properties they have to fulfill [3,4].

In recent years, organic-inorganic hybrid materials, particularly composite materials based on polymers and metal oxide nanoparticles, have attracted the attention of researchers [5]. The dispersion of nanoparticles in a polymer matrix conducts special materials that could own new properties characteristic to both components (nanoparticles and polymer), such as flexibility, tensile strength and biodegradability [6].

Some of the most used nano-composites used to treat wounds in the skin include zinc oxide, leading to a multifunctional material, due to its properties. Zinc oxide (ZnO) is non-toxic, has very good electrical and optical properties, is environmentally friendly, stable in reducing atmosphere, absorbs ultraviolet (UV) light, has antimicrobial properties, is a semiconductor and its price is very low [7]. Although the antibacterial activity of ZnO is well known, the mechanism is not completely clear, and some questions in that regard still requiring detailed explanations. Researchers have studied the morphological changes induced by ZnO nanoparticles to the bacteria by scanning electron microscopy (SEM), field emission scanning electron microscopy (FESEM) and transmission electron microscopy (TEM), trying to identify the antimicrobial mechanisms. Therefore, various mechanisms of action for ZnO nanoparticles against bacteria have been published, such as direct contact between the ZnO nanoparticles and the bacterial cell wall, release of antimicrobial ions, mainly Zn^2+^, and formation of reactive oxygen species (ROS) [8,9].

Antimicrobial dressings include those that incorporate an antiseptic agent, which is a biocide used to kill or inhibit the growth of microorganisms present in the wound or on intact skin. Recent advances in technology have led to the development of a large number of antiseptic products that are less harmful to healthy tissue while being extremely effective in pathogens colonization [1]. These antiseptics include silver, zinc oxide, titanium oxide, iodine. Dressings that incorporate such antiseptics can be successfully used to avoid microbial contamination [10,11,12].

The electrospinning technique was described by Rayleigh in 1897, and since then it was developed to obtain organized fibers [13]. This method is used to obtain non-woven fabrics, with extremely small fiber diameter, in the range of nanometers. The fibers have a large surface area and are easy to be functionalized for various purposes. Over 200 types of synthetic, natural and mixt polymers can be electrospun to obtain various types of fibers [14,15,16,17].

Polyvinyl alcohol (PVA) is a linear synthetic polymer produced by partial or complete hydrolysis of polyvinyl acetate. The degree of hydrolysis determines its physical and chemical properties. The resulting polymer is adaptable for many applications. It is commonly used in textile industry, paper manufacturing, food packaging and the medical field. Polyvinyl alcohol is used as a biomaterial due to its biocompatibility, as well as non-toxic and non-carcinogen properties [11,18].

Sodium alginate is a biomaterial used in numerous applications in tissue engineering and regenerative medicine fields, because of its properties such as biocompatibility, and the ease of gelling and structural similarity of the obtained gels to the extracellular tissue matrix [19]. 

In this paper, it was aimed to develop composite materials based on zinc oxide nanoparticles and polyvinyl alcohol and sodium alginate fibers, for the design of antimicrobial wound dressings. The nanoparticles were synthesized by the microwave assisted hydrothermal method or through the polyol method, which lead to various morphologies, depending on the synthesis parameters. The correlation between the synthesis parameters, antibacterial properties and in vitro testing results of the obtained composite materials were investigated. 

## 2. Materials and Methods 

### 2.1. Materials

Zinc chloride (ZnCl_2_, >98% purity, Mw = 136,30 g/mole), ammonium hydroxide solution (NH_4_OH, 25%), sodium hydroxide pellets (NaOH, >98% purity, Mw = 40 g/mole), sodium sulfate (Na_2_SO_4_, >98% purity, Mw = 142,04 g/mole), zinc sulphate (ZnSO_4_, >99% purity), zinc acetate dihydrate (Zn(CH_3_COO)_2_ · 2H_2_O, >98% purity, Mw = 219,51 g/mole), ethylene glycol (C_2_H_6_O_2_, >99.5% purity), ethanol (C_2_H_5_OH, >99.8% purity) and acetone (C_3_H_6_O, >99.9% purity) were used for preparing the zinc oxide (ZnO) powder. 

For the synthesis of fibers, sodium alginate (C_6_H_9_NaO_7_), polyvinyl alcohol PVA (>98% purity), glutaraldehyde (25%), ammonium hydroxide solution (25%), methanol (>99.9% purity), calcium chloride (CaCl_2_, >97% purity) and ethanol (>99.8% purity) were used.

### 2.2. ZnO Nanostructures Synthesis

In order to determine which process would lead to a better antimicrobial activity of zinc oxide, two synthesis methods were used—the microwave-assisted hydrothermal and polyol methods. 

In the microwave-assisted hydrothermal method, ZnCl_2_ and ZnSO_4_ were used as Zn^2+^ precursors. The general steps implied in this type of synthesis are as follows: Samples are weighed into vials, and suitable reagents are added. Vials are placed in a rack, which is automatically lowered into the reaction chamber. The chamber is sealed and pre-pressurized with inert gas, which physically acts as a cap for the vials, avoiding boiling of the solutions and preventing cross contamination. At the completion of the microwave run, a built-in cooling device rapidly lowers the temperature.

Firstly, if ZnSO_4_ is used as a precursor (synthesis H1), 6 mL of ammonia (25%) was mixed with distilled water to form 100 mL of solution. The mixture was added dropwise over 100 mL of 0.04 M Zn^2+^ precursor solution. The obtained mixture was placed in a Teflon vessel inside the autoclave, for a microwave-assisted hydrothermal treatment of 10 min at 120 °C and 7 bars pressure, then cooled to room temperature. Finally, the formed precipitate was collected by filtration, washed with distilled water twice and once with ethanol. The resulting powder was dried in a vacuum.

For the second synthesis of ZnO (H2), 60 mL of a 0.5 M aqueous solution of zinc chloride was added over 100 mL of 2 M NaOH solution. The obtained mixture was stirred for 15 min while maintaining pH to 12 (by adding NaOH solution, as needed). Following this, the solution was placed in an autoclave where it was maintained at a temperature of 90 °C and a pressure of 7 bars for 10 min. After the hydrothermal treatment, the obtained precipitate was collected by filtration, washed with distilled water twice and once with ethanol, then dried in the oven at 90 °C for 5 h [20].

The third synthesis (H3) was performed starting from 0.60 g of Zn(OH)_2_ previously prepared according to [21], and 50 mL of 2 M solution of Na_2_SO_4_ that were mixed with 50 mL of 4 M solution of NaOH. After stirring for one hour, the solution was transferred to a vial and introduced into the microwave-assisted hydrothermal reaction chamber for 10 min, at a temperature of 140 °C and a pressure of 7 bar. The obtained precipitate was filtered, washed with distilled water twice and once with ethanol and oven dried at 80 °C for 6 h.

In regards to the polyol synthesis, two different routes were used, as described below. Firstly (P1), zinc acetate was dissolved in ethylene glycol by refluxing at 160 °C to form a 1 M solution. After the precursor has been completely dissolved, the mixture was refluxed for further 12 h at the same temperature. The formed suspension was centrifuged at 32,000 rpm for 15 min using an ultracentrifuge in order to separate the nanoparticles of the supernatant. Subsequently, ZnO was washed twice with ethanol, followed by a new forced separation by centrifugation. The obtained powder was dried in the oven at 80 °C for 6 h [22]. 

The second polyol synthesis of ZnO nanoparticles (P2) started also from Zn(CH_3_COO)_2_ 2H_2_O as Zn^2+^ precursor. It was dissolved in 1,3-propanediol, then heated to 160 °C and maintained at this temperature for 1 h. At the end of the reaction, the precipitate was centrifuged, washed several times with acetone and ethanol and then dried in a vacuum oven at 50 °C for 12 h in order to obtain the ZnO nanopowder [23].

### 2.3. Composite Material Synthesis

A 2% Sodium alginate (SA) and polyvinyl alcohol (PVA) (16%) solutions were prepared, by dissolving 2 g of alginate, respectively 16 g of polyvinyl alcohol in deionized water. The SA/PVA solution was obtained by mixing both solutions in different (3:1, 1:1, 1:3) mass ratios, for 10 min. The optimal SA/PVA ratio for electrospinning, by means of viscosity of spinning solution, was found to be 1:1. ZnO nanoparticles previously prepared were added to the polymer solution so that the content of ZnO in the composite fibers (after electrospinning) to be 5% (wt.), depending on the SA and PVA quantities. The obtained suspension was sonicated and subjected to a magnetic stirring for 1 h. The equipment used for the fibers synthesis was a Tong LiTech Nanofiber Electrospinning Unit equipped with a Tong LiTech-TL-FG injectomat system with DC source (0–50 kV) (Shenzhen, China), equipped with 2 syringes of 20 mL with metallic needles, having 0.6 mm internal diameter. The collector was wrapped in a special paper to facilitate the fibers removal. The solutions were loaded into syringes and the pumping was performed at a rate of 0.5 mL/h. The tension applied to the needle was of 20 kV, at a distance of 5 cm from the collector [10,11].

Both polyvinyl alcohol and sodium alginate are poorly soluble in water, so their stability in water needs to be improved. The classic method of improving the stability properties of polyvinyl alcohol is to treat it with vapors of glutaraldehyde (2%) as well as with NH_3_ vapors. 

In our study, the combination of polyvinyl alcohol and sodium alginate requires a combination of cross-linking methods [24]. 

The obtained fibers were placed in a desiccator where they were subjected to NH_3_ vapor treatment for 48 h to crosslink the polyvinyl alcohol, and then introduced into a vacuum oven to remove unreacted NH_3_. For the crosslinking of sodium alginate, the fibers were introduced into a solution of CaCl_2_ (1%) in ethanol. This is done by trituration with methanol and CaCl_2_ solution, in order to replace Na^+^ ions with Ca^2+^ ions.

### 2.4. Structural and Morphological Characterization

X-ray diffraction analysis (XRD) was performed in order to characterize the synthesized powders from the point of view of their crystallinity as well as of the component phases. XRD analysis was performed using a Empyrean equipment from Malvern PANalytical, Bruno, Nederland in Bragg-Brentano geometry equipped with a Cu-anode (λCuKα = 1.541874 Å) X-ray tube with in-line focusing, programmable divergent slit on the incident side and a programmable anti-scatter slit mounted on the PIXcel3D detector on the diffracted side. The analysis was acquired on the 15–80° angle range, with the acquisition step of 0.02° and 100 s acquisition time per step.

The morphology of the obtained powders and composite fibers was studied via scanning electron microscopy (SEM), with a Quanta Inspect F50 microscope coupled with an energy dispersive spectrometer (EDAX) (Thermo Fisher (former FEI), Eindhoven, Nederland). 

### 2.5. Antimicrobial Assay

#### 2.5.1. Minimal Inhibitory Concentration (MIC) of the ZnO Nanopowders 

Strains of *Staphylococcus aureus* ATCC 25923; *Escherichia coli* ATCC 25922 and *Candida albicans* ATCC 10231 were purchased from the American Type Culture Collection (Virginia, USA) and maintained in the Lab as Glycerol stocks. Fresh cultures obtained in nutritive broth from glycerol stocks were inoculated on LB agar (for bacteria) or Sabouraud agar (for *C. albicans*) and incubated for 24 h at 37 °C to obtain cultures that were used for all subsequent studies. The antimicrobial activity of various zinc oxide nanoparticles has been studied by determining the minimum inhibitory concentration against *S. aureus*, *E. coli* and *C. albicans* laboratory strains. Minimal inhibitory concentrations were established by an adapted microtiter method [25]. Various concentrations (ranging 0.005—5 mg/mL) of the obtained nanoparticles were used to assess their effect against microbial growth, for 24h at 37 °C, in nutritive broth. MIC was considered as the lowest concentration to inhibit the microbial development. This was established by naked eye evaluation and spectroscopy measurement of microbial cultures at Abs = 600 nm.

#### 2.5.2. Growth Inhibition of the Obtained Dressings—Qualitative Assay

To evaluate the antimicrobial effect of the obtained dressings, an adapted version of the disc diffusion method was used. Microbial suspensions prepared in sterile saline buffer were obtained from each strain and adjusted to an optical density of 0.5 McFarland (1.5 × 10^8^ CFU (colony forming units)/mL). These were used to inoculate the entire surface of the nutrient agar Petri dishes. After inoculation, 6 mm size samples of the sterile coatings were aseptically deposited on the inoculated agar surface. Petri dishes containing the samples were incubated for 24 h at 37 °C to allow the growth of bacteria. After incubation, the diameter of growth inhibition zone (mm) was measured. A wider inhibition zone suggests a higher antimicrobial effect of the fibrous dressing.

### 2.6. Biocompatibility In Vitro 

#### 2.6.1. MTT Assay

The human mesenchymal amniotic fluid stem cells (AFSC) were used to evaluate the biocompatibility of ZnO nanoparticles. The cells were cultured in DMEM medium (Sigma–Aldrich, Missouri, MO, USA) supplemented with 10% fetal bovine serum, 1% penicillin and 1% streptomycin antibiotics (Sigma–Aldrich, Missouri, MO, USA). To maintain optimal culture conditions, the medium was changed twice a week. The biocompatibility was assessed using MTT assay (Vybrant^®^ MTT Cell Proliferation Assay Kit, Thermo Fischer Scientific, Massachusetts, MA, USA). The assay is a colorimetric method that allows quantitative assessment of proliferation, cell viability and cytotoxicity. The viable cells reduce yellow tetrazolium salt MTT (3- (4,5dimetiltiazoliu) -2,5-diphenyltetrazolium bromide) to a dark blue formazan via mitochondrial enzymes. Briefly, the AFSC were grown in 96-well plates, with a seeding density of 3000 cells / well in the presence of the analyzed samples (5 mg/mL concentration of each ZnO nanopowder) for 72 h. Then, 15 mL of Solution I was added and incubated at 37 °C for 4 h. Solution II was added and pipettes vigorously to solubilize formazan crystals. After 1 h, the absorbance was read using a spectrophotometer at 570 nm (TECAN Infinite M200, Männedorf, Switzerland).

#### 2.6.2. Evaluation of Cell Morphology and Viability by Fluorescence Microscopy

The biocompatibility of the obtained powder materials with AFSC was also evaluated based on fluorescence microscopy using RED CMTPX fluorophore (Thermo Fischer Scientific, Massachusetts, MA, USA), which is a cell tracker for long-term tracing of living cells. The CMTPX tracker was added in cell culture treated with ZnO nanoparticles and the viability and morphology of the AFSC was evaluated after 5 days. The CMTPX fluorophore was added in the culture medium at a final concentration of 5 μM, incubated for 30 min in order to allow the dye penetration into the cells. Next, the AFSC were washed with PBS and visualized by fluorescent microscopy. The photomicrographs were taken with Olympus CKX 41 digital camera driven by CellSense Entry software (Olympus, Tokyo, Japan).

## 3. Results

### 3.1. X-ray Diffraction (XRD)

**Figure 1:**
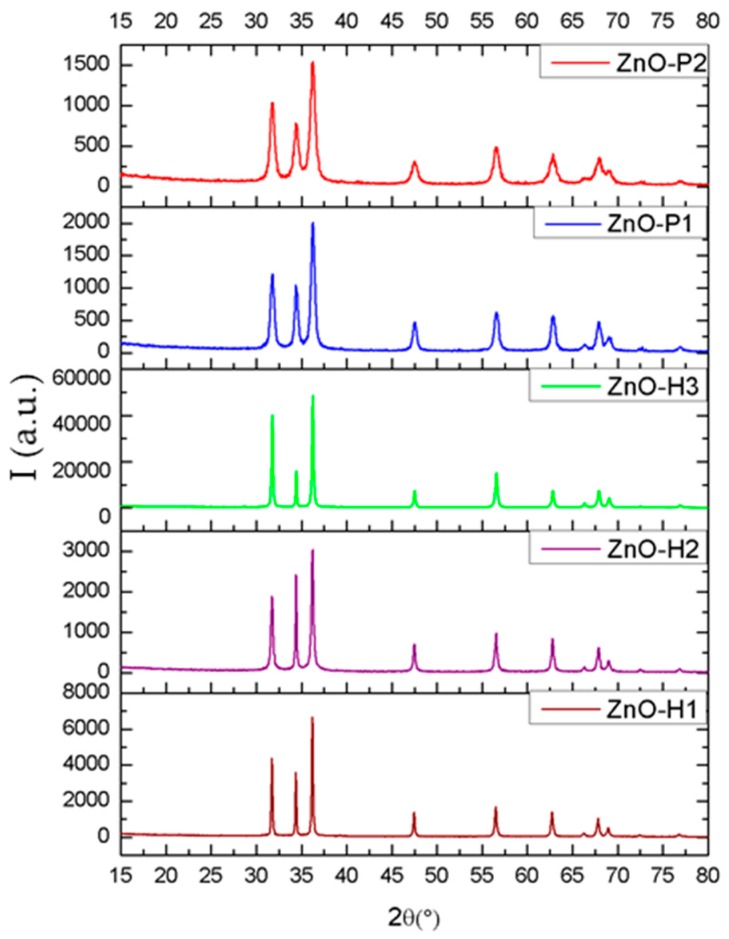
X-ray diffraction analysis (XRD) patterns of synthesized zinc oxide, obtained through the microwave assisted hydrothermal method (H1–H3), respectively through the polyol method (P1, P2).

The XRD patterns of ZnO powders prepared via the microwave assisted hydrothermal method and the polyol method are shown in Figure 1, with each pattern describing the powders obtained starting from different precursors, as described above. 

All synthesized ZnO powders are hexagonal, with a wurtzite structure belonging to the space group P63mc. The XRD patterns denote that the diffraction peaks position matches the reported values in PDF4+ card (no. 01-078-2585). The only contribution to the patterns is from ZnO, which clearly shows high purity products. Moreover, in the case of ZnO-H2 sample, the pattern reveals an oriented growth of the structure along the c-axis direction because the (002) reflection at 34.4° 2θ has a higher relative intensity as compared to the wurtzite structure.

Moreover, it can be noticed the broadening of the diffraction peaks, if the polyol method is used. In Table 1, the values for the crystallite size and micro strains, obtained by Rietveld refinement of the patterns, are shown. It can be easily seen that the crystallite size is varying inversely proportional with the micro strains, both being influenced by the preparation method and parameters. The values determined for the mean crystallite size is consistent with the finding above, that is, smaller sizes leading to a broadening of peaks. 

### 3.2. Scanning Electron Microscopy (SEM)

#### 3.2.1. Scanning Electron Microscopy on ZnO Nanopowders

**Figure 2:**
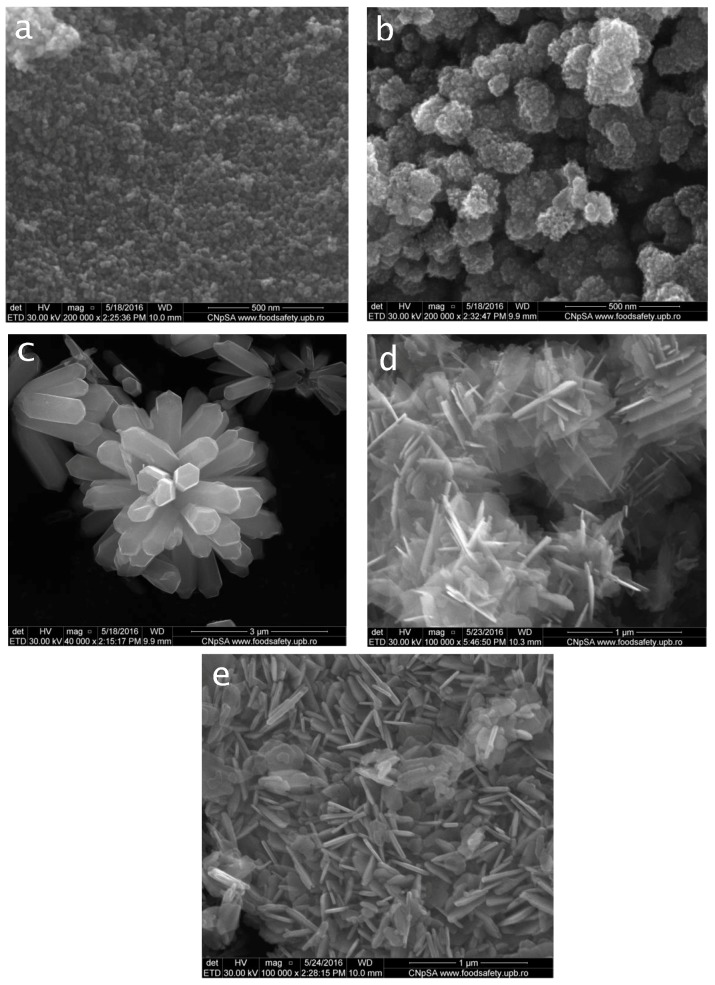
Scanning Electronic Microscopy (SEM) images of zinc oxide powder obtained by polyol ((**a**)—P1, (**b**)—P2) and hydrothermal ((**c**)—H1, (**d**)—H2, (**e**)—H3) methods.

Analyzing the SEM images presented in Figure 2, the effect of hydrothermal and polyol synthesis method, precursors and synthesis parameters on the morphology of zinc oxide nanostructures can be observed. 

Therefore, SEM images revealed different morphologies and sizes for the zinc oxide particles obtained via different synthesis methods. For the samples obtained by polyol method, spherical morphology was obtained, while for the powders obtained using microwave-assisted hydrothermal method, flower-type and plate like morphologies were identified. 

For P1 sample, prepared in ethylene glycol (Figure 2a), it can be observed that the nanopowder consists mostly of small spherical particles ranging from 10 to 15 nm in size, with a very narrow distribution. In the case of the second polyol synthetized nanoparticles P2 Figure 2b, the morphology changes from uniformly distributed nanoparticles to raspberry like aggregates ~100 nm in size, made up of assembled nanoparticles of small spherical particles ranging from 10 to 15 nm in size.

The SEM images show that the synthesized nanostructures via hydrothermal method are grown in large density and possess multilayered structure. Figure 2c illustrates flower like ZnO agglomerates, composed of prismatic form particles. The average diameter of the grown structures was determined in the range of 1–2 μm.

For the second type of nanostructures synthesized via microwave assisted hydrothermal method (Figure 2d), irregular morphologies constructed from ZnO nanosheets can be observed. These nanosheets are of about 20 nm in thickness. The morphology for the last sample (e) is similar to the H2 sample, with modified size of the plates, for these conditions being smaller (Figure 2e).

#### 3.2.2. Scanning Electron Microscopy on Composite Polymer/ZnO/Sodium Alginate Fibers

The polymer/ZnO fibers were obtained by the electrospinning method and it was observed that even at very low concentrations of sodium alginate, the conductivity had an appropriate value for fiber formation. By homogenizing the two polymer solutions (16% polyvinyl alcohol and 2% sodium alginate) in a 1:1 mass ratio, a balance was created between viscosity and conductivity, which resulted in smooth fibers formation, without large variations in their diameter. When zinc oxide was added to the solution, a slight increase in conductivity was found, which was favorable for the process. In Table 2, the properties of the solutions used for electrospinning were summarized.

**Figure 3:**
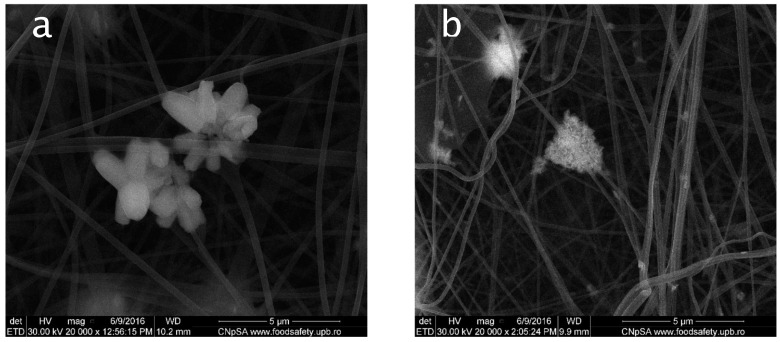

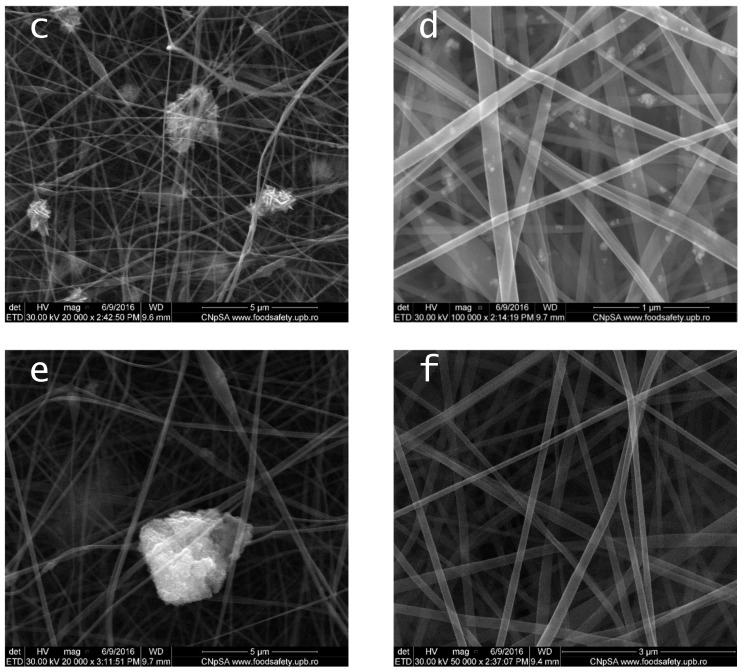
SEM images for composite fibres (**a**) PVA/SA/ZnO-H1, (**b**) PVA/SA/ZnO-H2, (**c**) PVA/SA/ZnO-H3 (**d**) PVA/SA/ZnO-P1, (**e**) PVA/SA/ZnO-P2, (**f**) PVA/SA.

In Figure 3 are presented SEM images realized on composites obtained via electrospinning technique. 

Figure 3 shows scanning electron microscopy images of polyvinyl alcohol and sodium alginate fibers containing 5% zinc oxide previously synthesized. All samples are characterized by a porous structure with a random orientation of fibers. The control sample, containing only polyvinyl alcohol and sodium alginate, has a smooth fibrous structure with fibers diameter of 140–190 nm, while the for the composite structures the fibers have a diameter between 160 and 220 nm. 

In the case of composites with ZnO nanostructures synthetized via H1, H2, H3, P2, due to the formation of zinc oxide aggregates and agglomerates larger than fibers diameters, there is no homogenous distribution of ZnO in the polymer fibers matrix. The only one that showed a good dispersion on the fiber structure was the zinc oxide particles obtained through the polyol method, starting from zinc acetate and ethylene glycol P1, which has a very uniform distribution along the fibers (Figure 3d).

### 3.3. Antimicrobial Tests

#### 3.3.1. Minimal Inhibitory Concentration of the ZnO Nanopowders

**Figure 4:**
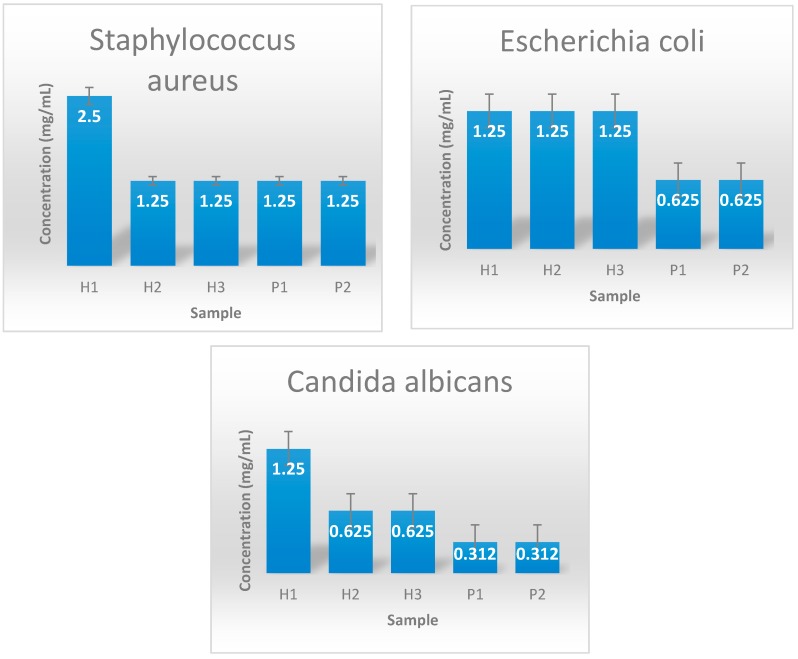
Minimal inhibitory concentration of zinc oxide samples for *S. aureus*, *E. coli* and *C. albicans*.

The minimum inhibitory concentration is defined as the lowest concentration which significantly inhibits the bacterial growth. All tested samples showed significant antibacterial activity. Most of the obtained MICs ranged 0.312–1.25 mg/mL. The best results were obtained for zinc oxide nanoparticles synthesized by the polyol method. These results can be attributed to differences in shape and size of the obtained nanoparticles, but also the differences in preparation. Figure 4 shows the minimum inhibitory concentrations of synthesized zinc oxide against *S. aureus*, *E. coli* and *C. albicans* strains.

#### 3.3.2. Evaluation of Growth Inhibition of the Obtained Coatings

**Figure 5:**
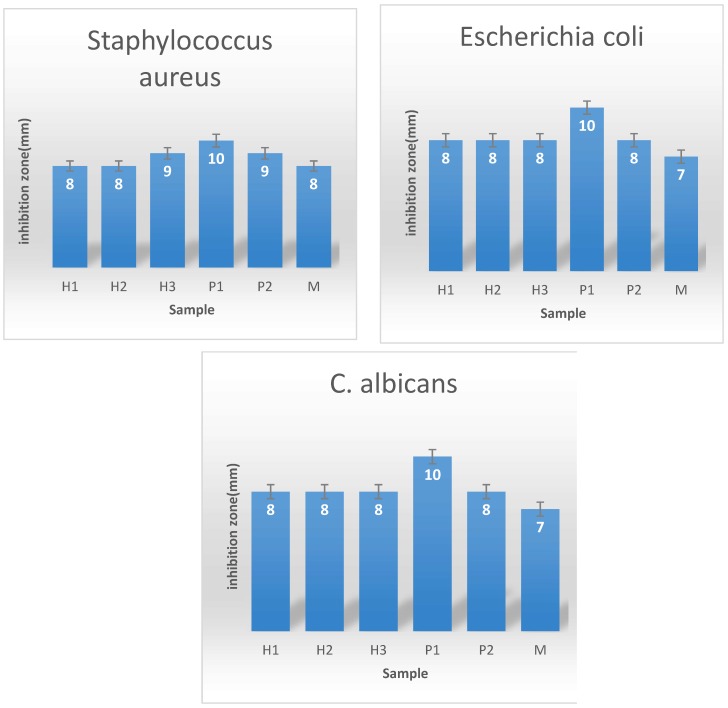
Growth inhibition zone for *S. aureus*, *E. coli* and *C. albicans* strains developed in the presence of the electrospinning obtained coatings, containing ZnO nanoparticles prepared by various microwave-assisted hydrothermal (H1–H3) and polyol methods (P1, P2). M represents the electrospinning fibers without ZnO nanoparticles.

The evaluation of growth inhibition zone is one of the most utilized tests when characterizing the antimicrobial potential of a solid bioactive material, a coating or dressing. This assay allows for the determination of the ability of the material to release antimicrobial doses of the antimicrobial drug (or nanoparticles). Figure 5 shows the antimicrobial activity of the obtained hybrid materials tested against *E. coli* Gram negative bacteria, Gram positive *S. aureus* and *C. albicans* fungi. As it can be easily observed, these results correlate with the MIC test data, as the fibrous dressings containing ZnO nanoparticles designed by the polyol method showed the greatest values of the growth inhibition zones, being thus the most efficient materials (Figure 5). 

Antimicrobial tests demonstrated that all the designed fibers could manifest antimicrobial effect and their efficiency is slightly different among the tested strains. These differences may be explained by the morphological particularities of the microbial species utilized in the study, the main differences being at the level of cellular wall. This hypothesis is supported also by previous data showing nanostructured materials may have different influence on Gram positive, Gram negative and fungal microorganisms [26].

### 3.4. Biocompatibility

#### 3.4.1. In Vitro MTT assay

**Figure 6:**
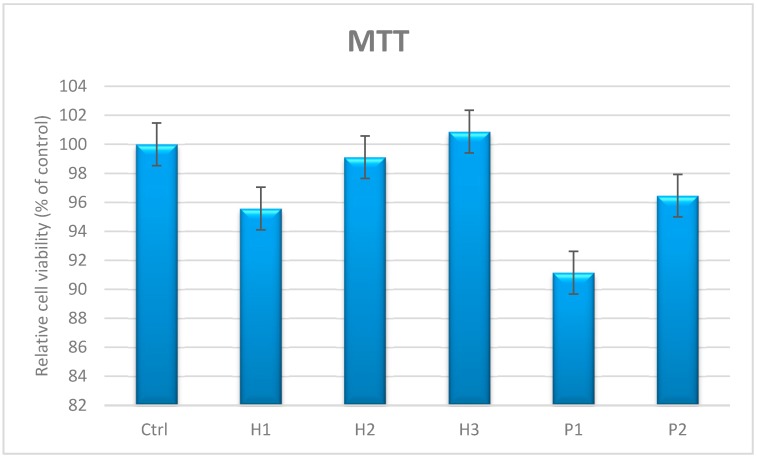
AFSC viability in the presence of ZnO nanoparticles.

In Figure 6, the results are presented of the cell viability test for H1, H2, H3, P1 and P2, and also for the untreated control. The highest cell viability was observed in the presence of H3 samples, where the cells proliferate more than the control, while the lowest viability was recorded for P1, with 92% compared to control cells. Despite low differences in the viability degree, these differences are not of significant biological value since all the samples showed very good viability over 90% compared to control cells, suggesting that ZnO nanoparticles are biocompatible with human AFSC. 

MTT test demonstrates no significant difference in the viability of AFSC cultured in the presence of ZnO nanoparticles as compared with untreated control. Results are represented as mean ± standard error, n = 3, * p < 0.05 (T test).

#### 3.4.2. In Vitro–Evaluation of Cell Morphology and Viability by Fluorescence Microscopy

**Figure 7:**
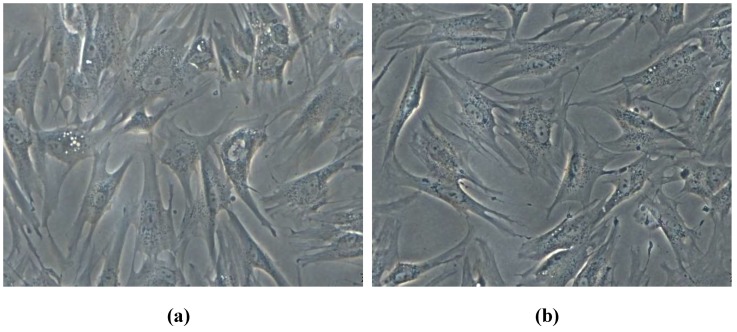

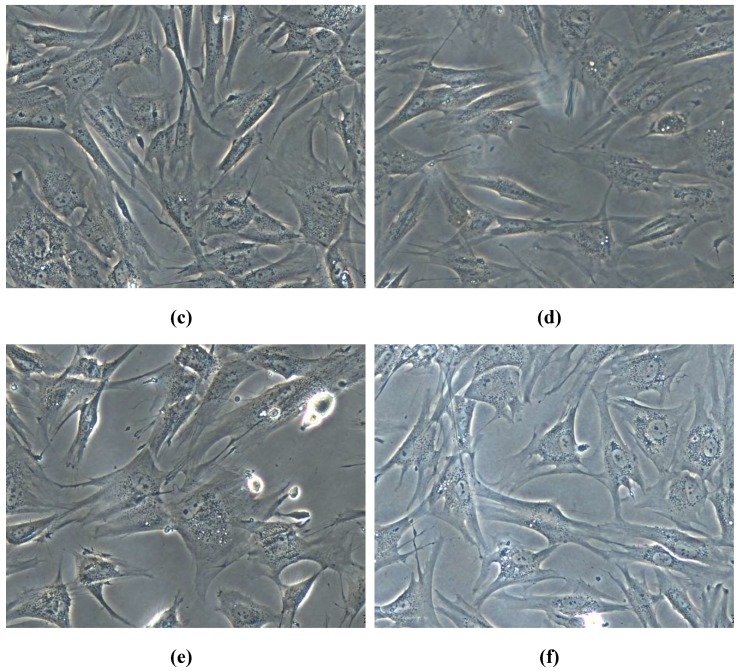
Optical microscopy images of human amniotic fluid stem cells (AFSC) cultures developed at 3 days in the presence of the obtained ZnO nanomaterials H1 (**a**), H2 (**b**), H3 (**c**) and P1 (**d**), P2 (**e**) and cell control (**f**).

The morphology and overall aspect of the diploid cultured human cells can be observed in Figure 7.

By comparing the results for ZnO nanoparticles containing samples with the control one, it can be concluded that the cell morphology was not modified in the presence of ZnO nanoparticles. 

Figure 7 shows representative photomicrographs of human AFSC treated with ZnO nanoparticles for 72 h. The AFSC present a normal fibroblast-like phenotype, with numerous filopodia, suggesting that the cells are viable with an active metabolism and well attached on the substrata. The presence of these pseudopodia is due to actin cytoskeleton, an important protein involved in cell interactions and migration. 

## 4. Discussion

The degree of crystallinity and the size of the crystals can be estimated from the width of the diffraction peaks, which in this case varies depending on the synthesis method and the conditions used. Thus, the highest degree of crystallinity was obtained by the hydrothermal method starting from Zn(SO_4_), and the lowest by the polyol method using 1,3-propanediol as the solvent.

The spherical morphology observed through SEM analysis for ZnO samples obtained by using the polyol method may be possible due to the coordination between ethylene glycol / 1,3-propanediol and Zn^2+^ molecules or to selective anchoring of solvent molecules on ZnO crystals, both favoring the production of spherical morphology. Both ethylene glycol and 1,3-propanediol contain two hydroxyl groups in the molecule, which could inhibit preferential growth in the, c, direction, leading to spherical morphology. The low particle size can be attributed to the spontaneous formation of Zn(OH)_2_ monomers resulting from the reaction between zinc acetate and solvent, which results in a higher nucleation rate. Therefore, more particles are formed, which means smaller sizes of the particles, in correlation with higher concentration of the initial nuclei.

For ZnO samples obtained via microwave-assisted hydrothermal method, the possible mechanism of formation of flower-type morphologies can be explained by the decomposition of zinc chloride and ammonia at the reaction temperature. The concentration of Zn^2+^ and OH ions gradually increases and ZnO nuclei begin to form when the degree of over-saturation exceeds the critical value. The concentration of NH_4_OH plays a vital role in the formation of the flower structures. High values mean that the supplied OH^−^ ions are enough for the zinc oxide nucleation, while for too low concentrations irregular or rod type morphologies would have been obtained.

By correlating X-ray diffraction with scanning electron microscopy results, it can be stated that in the samples obtained via microwave-assisted hydrothermal method, a preferential increase is observed in the direction of the, c, axis. This increase can be attributed to the high temperature at which the reaction takes place (140 °C).

For the polymer/ZnO fibers, the SEM analysis revealed a porous structure and a random orientation of fibers. Due to the formation of aggregates and agglomerates which sometimes are larger than the diameter of the fibers, zinc oxide nanoparticles are not fully incorporated into the structure, resulting in less uniform deposition of nanoparticles on fibers. A homogeneous dispersion of oxide nanoparticles in the fiber structure could be obtained only for those prepared through the polyol method, starting from zinc acetate and ethylene glycol. 

In vitro experiments revealed that all nanostructured fibrous coatings have a significant antimicrobial activity, their effect being influenced by the ZnO nanoparticle concentration and the synthesis method. Both qualitative (diameter of growth inhibition zone) and quantitative (MIC assay) tests proved that samples containing ZnO nanoparticles have the greatest antimicrobial effects, although sodium alginate may potentate the antimicrobial activity of the coating, since it seems to have an intrinsic antibacterial activity [27]. Their high antimicrobial effect was correlated with a very good in vitro biocompatibility, as revealed by the microscopy evaluation and MTT assay. 

It seems that the use of ZnO nanoparticles improves acute and chronic wounds healing due to antibacterial, anti-inflammatory and increased re-epithelialization properties [28]. The mechanisms of action of ZnO nanoparticles involves its participation as a cofactor in the enzymatic complexes that promote migration of keratinocytes and the implication in the formation of reactive oxygen species which penetrate the bacterial cell membrane [29]. One of the main conditions of drug therapy is to be non-toxic to the host cells. The viability of cells in the presence of ZnO nanoparticles is influenced by particle sizes and concentration. ZnO nanoparticles with a smaller size (higher specific surface areas) showed highest cellular viability in vitro [30]. Also, low concentrations of ZnO nanoparticles are nontoxic to eukaryotic cells, according to recent results [31]. Pati et al. (2015) showed that ZnO nanoparticles at the bactericidal dose have no detrimental effects on monocytes cell lines. Our results showed that ZnO nanoparticles synthetized by hydrothermal and polyol methods present no cytotoxicity on human amniotic fluid derived stem cells (AFSC) [32]. 

The obtained results correlate with recent literature demonstrating the antimicrobial effect of ZnO nanoparticles and their utility for biomedical applications [33], and highlights that electrospinning may be an efficient technique to obtain coatings and fibrous materials with multiple applications. The discussed properties recommend the designed nanostructured fibrous coatings for biomedical applications, suggesting they could be efficiently used for wound healing, being able to avoid microbial colonization and multiplication of the wound, which is one of the main causes of complication and delayed healing in chronic wounds [34]. 

## 5. Conclusions

This study reports on the design and characterization of a new type of antimicrobial dressings based on polyvinyl alcohol, sodium alginate and zinc oxide nanoparticles. ZnO nanoparticles were synthesized by the microwave assisted hydrothermal and polyol methods, using different synthesis parameters and Zn^2+^ precursors. It was observed that the synthesis approach impacts on the subsequent biological effects of ZnO nanoparticles, due to different morphology and size of powders.

The resulting powders were characterized by X-ray diffraction that demonstrated the production of zinc oxide in hexagonal form. The sample morphology was analyzed by scanning electron microscopy that showed different microstructure of particles, for different processing parameters. Thus, flower-like shaped agglomerates, plates, rods and cvasi-spherical nanoparticles were obtained. Polymeric fibers were obtained by electrospinning and Zinc oxide powders were used to enhance their antimicrobial properties.

The nanopowders and fibers have shown significant antimicrobial activity, being efficient against Gram positive (*S. aureus*), Gram negative (*E. coli*) and opportunistic yeast (*C. albicans*) models. By correlating the dimensions and morphologies of the obtained particles with their antimicrobial action, it can be concluded that zinc oxide with the smallest particle size exhibited the best activity, as expected. As for the composite materials, the high degree of dispersion of ZnO-P1 in the structure has led to remarkable improvement in their antimicrobial properties. Cellular viability tests confirm that the addition of 5% (wt.) ZnO nanopowder to the obtained fibers does not induce any cytotoxic effects in vitro, with the tested diploid cells presenting a normal morphology and development in the presence of the designed coatings.

## Figures and Tables

**Table 1:** Mean crystallite size and micro-strains determined from the XRD patterns for ZnO powders.

	ZnO-H1	ZnO-H2	ZnO-H3	ZnO-P1	ZnO-P2
Mean crystallite size (nm)	40.47 ± 16.22	15.71 ± 1.59	26.78 ± 2.80	14.63 ± 0.47	9.58 ± 1.35
Micro strain (%)	0.23 ± 0.02	0.56 ± 0.15	0.33 ± 0.08	0.60 ± 0.18	0.90 ± 0.16

**Table 2:** Main properties of solutions for electrospinning.

Solutions	Concentration, %	Viscosity, cPs	Conductivity, mS
**Polyvinyl Alcohol**	16	586	0.465
**Sodium Alginate**	2	61	3.579
**Polyvinyl Alcohol / Sodium Alginate**	16/2	350	0.961
**PVA/SA/ZnO**	16/2/5	407	1.623

## References

[1] Aderibigbe B.A., Buyana B. (2018). Alginate in Wound Dressings. Pharmaceutics.

[2] Baker M.I., Walsh S.P., Schwartz Z., Boyan B.D. (2012). A review of polyvinyl alcohol and its uses in cartilage and orthopedic applications. J. Biomed. Mater. Res. Part B Appl. Biomater..

[3] Zhou Z., Yan D., Cheng X., Kong M., Liu Y., Feng C., Chen X. (2016). Biomaterials based on N, N, N-Trimethyl chitosan fibers in wound dressing applications. Int. J. Biol. Macromol..

[4] Manouchehri S. (2017). Can regenerative medicine and nanotechnology combine to heal wounds? The search for the ideal wound dressing. Nanomedicine.

[5] Nidhi K., Indrajeet S., Khushboo M., Gauri K., Sen D.J. (2006). Microstructural Imaging of Early Gel Layer Formation in HPMC Matrices. J. Pharm. Sci..

[6] Marius R., Andronescu E., Holban A.M., Vasile B.S., Iordache F., Mogos G.D., Chifiriuc M.C. (2016). Antimicrobial Nanostructured Bioactive Coating Based on Fe_3_O_4_ and Patchouli Oil for Wound Dressing. Metals.

[7] Chaturvedi A., Bajpai A.K., Bajpai J.K., Singh S. (2016). Evaluation of poly (vinyl alcohol) based cryogel–zinc oxide nanocomposites for possible applications as wound dressing materials. Mater. Sci. Eng. C.

[8] Manuscript A. (2013). Alginate: properties and biomedical applications. Prog. Polym. Sci..

[9] Stanković A., Dimitrijević S., Uskoković D. (2013). Influence of size scale and morphology on antibacterial properties of ZnO powders hydrothemally synthesized using different surface stabilizing agents. Colloid. Surf. B Biointerfaces.

[10] Kolodziejczak-Radzimska A., Jesionowski T. (2014). Zinc oxide-from synthesis to application: A review. Materials.

[11] Sirelkhatim A., Mahmud S., Seeni A., Kaus N.H.M., Ann L.C., Bakhori S.K.M., Hasan H., Mohamad D. (2015). Review on Zinc Oxide Nanoparticles: Antibacterial Activity and Toxicity Mechanism. Nano-Micro Lett..

[12] Adlhart C., Verran J., Azevedo N.F., Olmez H., Keinänen-Toivola M.M., Gouveia I., Melo L.F., Crijns F. (2018). Surface modifications for antimicrobial effects in the healthcare setting: a critical overview. J. Hosp. Infect..

[13] Kamoun E.A., Kenawy E.-R.S., Chen X. (2017). A review on polymeric hydrogel membranes for wound dressing applications: PVA-based hydrogel dressings. J. Adv. Res..

[14] Padmavathy N., Vijayaraghavan R. (2008). Enhanced bioactivity of ZnO nanoparticles—an antimicrobial study. Sci. Technol. Adv. Mater..

[15] Greiner A., Wendorff J.H. (2007). Electrospinning: A fascinating method for the preparation of ultrathin fibers. Angew. Chem. Int. Ed..

[16] Kim Y.-J., Matsunaga Y.T. (2017). Thermo-responsive polymers and their application as smart biomaterials. J. Mater. Chem. B.

[17] Gyles D.A., Castro L.D., Silva J.O.C., Ribeiro-Costa R.M. (2017). A review of the designs and prominent biomedical advances of natural and synthetic hydrogel formulations. Eur. Polym. J..

[18] Haider A., Haider S., Kang I.-K. (2018). A comprehensive review summarizing the effect of electrospinning parameters and potential applications of nanofibers in biomedical and biotechnology. Arab. J. Chem..

[19] Tijing L.D., Ruelo M.T.G., Amarjargal A., Pant H.R., Park C.-H., Kin D.W., Kim C.S. (2012). Antibacterial and superhydrophilic electrospun polyurethane nanocomposite fibers containing tourmaline nanoparticles. Chem. Eng. J..

[20] Kuen Y.L., Mooney D.J. (2000). Alginate: Properties and Biomedical Applications. Prog. Polym. Sci..

[21] Dawson W.J. (1988). Hydrothermal synthesis of advanced ceramic powders. Am. Ceram. Soc. Bull..

[22] Meshesha B.T., Barrabés N., Medina F., Sueiras J.E. (2009). Polyol mediated synthesis & characterization of Cu nanoparticles: Effect of 1-hexadecylamine as stabilizing agent. Nanotechnology.

[23] Liang S., Zhu L., Gai G., Yao Y., Huang J., Ji X., Zhou X., Zhang D., Zhang P. (2014). Synthesis of morphology-controlled ZnO microstructures via a microwave-assisted hydrothermal method and their gas-sensing property. Ultrason. Sonochem..

[24] Vasile O.R., Serdaru I., Andronescu E., Truşcă R., Surdu V.A., Oprea O., Ilie A., Vasile B.Ş. (2015). Influence of the size and the morphology of ZnO nanoparticles on cell viability. Comptes Rendus Chim..

[25] Yang L., Wang J., Xiang L. (2015). Hydrothermal synthesis of ZnO whiskers from ε-Zn(OH)2in NaOH/Na2SO4solution. Particuology..

[26] Zhang Y., Yang Y., Tang K. (2007). Physicochemical Characterization and Antioxidant Activity of Quercetin-Loaded Chitosan Nanoparticles Yuying. Polym. Polym. Compos..

[27] Pati R., Mehta R.K., Mohanty S., Padhi A., Sengupta M., Vaseeharan B., Goswami C., Sonawane A. (2014). Topical application of zinc oxide nanoparticles reduces bacterial skin infection in mice and exhibits antibacterial activity by inducing oxidative stress response and cell membrane disintegration in macrophages. Nanomed. Nanotechnol. Biol Med..

[28] Liakos I.L., Grumezescu A.M., Holban A.M., Florin I., Autilia F.D., Carzino R., Bianchini P., Athanassiou A. (2016). Polylactic Acid—Lemongrass Essential Oil Nanocapsules with Antimicrobial Properties. Pharmaceuticals.

[29] Slavin Y.N., Asnis J., Häfeli U.O., Bach H. (2017). Metal nanoparticles: Understanding the mechanisms behind antibacterial activity. J. Nanobiotechnol..

[30] Han Y., Yu M., Wang L. (2018). Physical and antimicrobial properties of sodium alginate / carboxymethyl cellulose fi lms incorporated with cinnamon essential oil. Food Packag. Shelf Life.

[31] Daghdari S.G., Ahmadi M., Saei H.D., Tehrani A.A. (2017). The effect of ZnO nanoparticles on bacterial load of experimental infectious wounds contaminated with Staphylococcus aureus in mice. Nanomed. J..

[32] Mihai M.M., Preda M., Lungu I., Gestal M.C., Popa M.I., Holban A.M. (2018). Nanocoatings for Chronic Wound Repair—Modulation of Microbial Colonization and Biofilm Formation. Int. J. Mol. Sci..

[33] Li L., Fernández-cruz M.L., Connolly M., Conde E., Fernández M., Schuster M., María J. (2015). Science of the Total Environment The potentiation effect makes the difference: Non-toxic concentrations of ZnO nanoparticles enhance Cu nanoparticle toxicity in vitro. Sci. Total Environ..

[34] Kalpana V.N., Rajeswari V.D. (2018). A Review on Green Synthesis, Biomedical Applications, and Toxicity Studies of ZnO NPs. Bioinorg. Chem. Appl..

